# Working for food is related to range use in free-range broiler chickens

**DOI:** 10.1038/s41598-021-85867-2

**Published:** 2021-03-18

**Authors:** Vitor Hugo Bessa Ferreira, Arthur Simoni, Karine Germain, Christine Leterrier, Léa Lansade, Anne Collin, Sandrine Mignon-Grasteau, Elisabeth Le Bihan-Duval, Elodie Guettier, Hélène Leruste, Ludovic Calandreau, Vanessa Guesdon

**Affiliations:** 1 JUNIA ISA, Comportement Animal et Systèmes d’Elevage, 48 Boulevard Vauban, BP 41290, 59046 Lille Cedex, France; 2 INRAE, CNRS, IFCE, Centre Val de Loire UMR Physiologie de la Reproduction et des Comportements, Université de Tours, 37380 Nouzilly, France; 3 INRAE, UE EASM, Le Magneraud, CS 40052, 17700 Surgères, France; 4 INRAE, Université de Tours, BOA, 37380 Nouzilly, France

**Keywords:** Animal behaviour, Behavioural methods

## Abstract

When animals prefer to make efforts to obtain food instead of acquiring it from freely available sources, they exhibit what is called contrafreeloading. Recently, individual differences in behavior, such as exploration, were shown to be linked to how prone an individual may be to contrafreeload. In this work, our main objective was to test whether and how individual differences in range use of free-range broiler chickens (*Gallus gallus domesticus*) were related to the individual motivation to contrafreeload. We also verified whether other behavioral variations could relate to range use. To that aim, over three different periods (before range access, first weeks of range access, and last weeks of range access), chickens with different ranging levels (low and high rangers) were submitted to a contrafreeloading test and had different behaviors recorded (such as foraging, resting, locomotion) in their home environment. During the contrafreeloading test, chickens were conditioned to one chamber presenting a foraging substrate and mealworms, while in the other chamber, mealworms were freely available on the floor. During testing trials, chickens had access to both empty chambers, and the time spent in each chamber was quantified. On average, low rangers preferred the chamber where mealworms were easily accessible (without the foraging substrate), while high rangers preferred the chamber where mealworms were accessible with difficulty, showing greater contrafreeloading. Out of ten behaviors recorded in chickens' home environment, foraging was the only one that differed significantly between our two ranging groups, with low rangers foraging, on average, significantly less than high rangers. These results corroborate previous experiences suggesting that range use is probably linked to chickens' exploratory trait and suggest that individual differences in free-range broiler chickens are present even before range access. Increasing our knowledge of individual particularities is a necessary step to improve free-range chicken welfare on the farm.

## Introduction

When animals prefer to make efforts to obtain food instead of acquiring it from freely available sources, they exhibit what is called contrafreeloading. In experimental tests, an individual animal can choose to press a lever several times for having access to food instead of choosing the free feed right next to the feed dispenser or, during a choice test with two chambers, spend more time on the side where the food is difficult to access than on the side where food is easily accessible^1,2^.

Contrafreeloading has been observed in a wide range of species, including humans^3^, and may seem, at first glance, counterintuitive. Indeed, according to theories of optimal foraging, learning, and motivation, animals should maximize their earnings and minimize energy expenditure (least effort hypothesis)^4^. However, the 'information primacy' model predicts that, under certain conditions, individuals will invest time and energy to collect as much information as possible from their environment, which could reduce costs in the long run through a more efficient feeding^5,6^. Contrafreeloading can therefore constitute a potential evolutionary advantage for the individuals exhibiting it. For example, Mongolian gerbils (Meriones unguiculatus) prefer to forage from difficult-to-access food sources when these are hidden. Otherwise, if easy and difficult available foods are visible, they prefer the easily accessible food^7^. Another theory suggests that contrafreeloading occurs because the behavioral responses requited to obtain food, e.g., foraging, are rewarding in their own right^5^. However, it does not seem to be the case: when provided with a choice between free food and making a response that no longer produces food, animals choose the free food^5^. Similarly, pigs (*Sus scrofa domesticus*) prefer a chamber with straw and hidden food over a chamber with straw only^2^.

Rearing conditions and selective pressures are known to modulate the individual motivation to contrafreeload^5^. Junglefowl, the modern chicken's ancestors, are more prone than domestic laying hens to make efforts to find food^8,9^. Domestic layers are, in turn, more prone to contrafreeload than standard broiler chickens^6^. The differences in contrafreeloading within this same species (*Gallus gallus*) facing different selection pressures (natural selection, selection for egg production, and selection for meat production, respectively) can be explained through resource theory allocation^6^. This theory predicts that when selection promotes certain expensive behaviors or biological processes, the energy for other demanding behaviors/processes must decrease. In domestic fowl*,* the need to forage or collect environmental information may be less important than the need to direct energy for reproduction and growth, which can significantly reduce contrafreeloading^10^.

The first study on contrafreeloading in domestic chickens mentions the existence of "significant and consistent differences" in how prone the tested animals were to access a difficultly-accessible food source^11^. Researchers demonstrated that in other farmed animals, such as in young cows, the willingness to exhibit contrafreeloading (by pushing a weighted door to obtain grain and hay) was linked to their exploratory behavior. The most exploratory cows lifted heavier maximum weights to have access to feed^12^. Therefore, individual differences in contrafreeloading may be linked to the way individuals perceive and interact with their physical and social environment.

Multiple studies have shown that free-ranging broiler chickens may vary significantly in their motivation to access the range, with some individuals ranging more than others^13,14^. The fact that some chickens are motivated to exit the barn and go outdoors for ranging and foraging, despite the indoor presence of everything necessary for their growth and surviving (feed, water, and conspecifics in predictable ways^15,16^), suggests that these individuals are, among other things, more prone to make efforts for feed than those who stay in the barn^17^. Therefore, free-range broiler chickens may be an appropriate model to study the relationship between their ranging behavior and the contrafreeloading phenomenon.

Much is known on the cognitive and perceptual abilities of chickens^18–20^. From the first days of life, chicks are capable of impressive features. They can perceive visual illusions^21^, complete mentally partially occluded objects^22^, form mental representations of disappearing objects^23^, and locate themselves using geometrical, position-specific, and non-geometrical cues^24–27^. Besides, they can also use ordinal cues (sense ordinality)^28,29^. Chicks are also know to present proto-arithmetic capacities^30^ and represent abstract proportional information from discrete elements^31^. They spontaneously discriminate possible and impossible objects^32^, prefer face‐like stimuli^33^, and prefer to approach biological motion patterns^34,35^. Far less research in chickens tried, however, to interconnect this basic knowledge to more applied questions^36^.

In the current work, using the free-range chicken as a model to study the contrafreeloading phenomenon, we had three main objectives. The first one was to assess whether and how contrafreeloading (i.e., to prefer an easy over difficult access to food, and vice-versa) under individual testing conditions would vary between chickens with different ranging behavior patterns (low and high rangers) and between different age periods. To that end, over three different periods of the chickens' production cycle: before the range access, during early range access (first weeks of access), and during late range access (last weeks of access), animals were subjected to a conditioned place preference task, where they were initially conditioned to associate the environmental information to a particular reward: a chamber with free mealworms or a chamber where mealworms were among a foraging substrate. During the test, chickens were given access to both empty chambers and were expected to show some preference for one of the two chambers through the time spent in each compartment. If an individual chose to spend more time in the chamber with difficult access to food (with the foraging substrate), it was considered a sign of contrafreeloading^2,6,37^. We hypothesized that free-range chickens' contrafreeloading behaviors would be linked to ranging behavior: low rangers would contrafreeload less, while high rangers, on the other hand, would contrafreeload more. Contrafreeloading was also expected to decrease over time since, according to the theory of resource allocation, animals would need to reduce the time spent on costly behaviors, such as foraging, and invest more energy in less costly behaviors, such as resting^38^.

In the barn and in the range, chickens' foraging behavior is considered a natural form of contrafreeloading^17^. The second objective of this work was, therefore, to measure chickens' foraging behavior in their home environment and during the three already mentioned periods of their life cycle. This was done to examine if this behavior follows the same pattern and hypothesis of contrafreeloading under the individual test situation: the time allocated to foraging would differ between low and high rangers, and foraging would decrease over time.

Finally, the third objective of this work was to test whether differences between low and high rangers were specific to contrafreeloading and foraging behaviors or whether there were other behavioral variations between these two groups and between different age periods. To that end, behaviors such as maintenance behaviors (feeding and drinking in the barn), locomotion, resting, comfort, and social behaviors, were also recorded. Based on the recent literature of free-range laying hens^17,39^, we expected low rangers to rest more and do more comfort behaviors than high rangers. Contrary to foraging and contrafreeloading, resting should increase over time in both groups, which would support the theory of resource allocation^6,38^.

## Methods

### Ethical statement

This study was conducted at the experimental unit UE 1206 EASM of INRAE, France, from October to December 2019 (fall). It was conducted under the INRAE ethics committee approval (APAFIS #21240-2019061811063005 v3) in agreement with the French legislation. This study was carried out in accordance with relevant guidelines and regulations, and in compliance with the ARRIVE guidelines.

### Animals and housing

The flock was composed of two hundred naked-neck (S757N) male broiler chickens (*Gallus gallus domesticus*) reared from their first day of life in a free-range system with a stocking density of 8 individuals/m^2^ in the barn (4.85 m × 5.15 m). The number of 200 birds has been decided in order to obtain an outdoor density corresponding to the requirements for French ‘Label Rouge’ chickens (i.e., ~ 2 m^2^/animal).

Continuous artificial lighting was provided during the first 3 days after placement; then, from day 4 to day 14, it was gradually decreased until there was a total use of natural lighting. The indoor ambient temperature was maintained at 28 °C during the first week and decreased by 1 °C each week until it reached 23 °C when the birds were 38 days old^40–43^.

On day 7, all 200 chicks were individually marked with a unique wing tag. A group of 117 individuals was randomly selected and identified via a rectangular yellow plastic poncho around the neck with unique acronyms for easy identification. Our previous work showed that, with this sample, we can have both a representative sample of the whole population and significant differences in range use between the extreme individuals (high and low rangers, see below)^40–43^.

Birds had indoor ad libitum access to food and water. The chickens had free access to the range at 36 days of age. The stocking density in the range was 0.4 individuals/m^2^ (27.3 m × 18 m). The range was a meadow-like, open space with vegetal cover, without trees or shelters available^40–43^.

### Behavioral observations (home environment condition)

Under the home environment condition (in the barn and in the range), one experimenter observed the behavior of 58 randomly selected individuals, among the 117 identified chickens, over three different periods: from day 14 to day 19 (period 1, before range access), from day 37 to day 46 (period 2, first weeks of range access), and from day 63 to day 72 (period 3, last weeks of range access).

To prevent any impact of the observer's presence and the noises linked to the stopwatch, the observer spent 3 h at least per day in the barn with the chicks for their 7 first days of life.

Due to time constraints, observation days were continuous over period 1, while for periods 2 and 3, the observations were executed 3 days per week, spaced of 1 or 2 days. Each observation day started between 8:00 and 9:30 in the morning and ended by 17:00.

Behavioral data were collected using the continuous sampling method, and each focal animal was observed for 30 s, four times a day. As days shortened over time, observations within the same day were spaced from 3 h (period 1) to 1 h and a half (periods 2 and 3).

The behaviors were divided into states and events. States were all behaviors lasting more than 1 s, and events those lasting less than 1 s. The events were recorded in 'all occurrences', meaning that every event behavior was counted within the 30 s of each focal animal. The observer recorded the duration of the state behaviors using a stopwatch and the number of occurrences of the event behaviors. The full ethogram is described in Table 1.

**Table 1:** Ethogram of recorded behaviors.

Traits	ATOL references^b^	Descriptions
**State behaviors**		
Standing	ATOL_0000835 (standing)	Stands in an upright position, with no foot movements
Resting	ATOL_0000837 (lying) ATOL_0000816 (immobility)	Sits relaxed, sleeps. The body and hocks are touching the floor
Locomotion	ATOL_0000805 (walking behavior)	Moves for at least two or more steps without pecking or scratching the ground
Foraging	ATOL_0000844 (investigative behavior trait) ATOL_0000845 (physical investigation)	Scratches and pecks the ground and/or a plant
Feeding/drinking	ATOL_0002158 (ingestion) ATOL_0000363 (feeding behavior trait) ATOL_0000361 (drinking behavior trait)	Include two behaviors: Feeding: Feeds at the feed containers in the barn Drinking: Drinks at the water containers in the barn
Social negative behaviors^a^	ATOL_0000902 (agonistic behavior) ATOL_0000813 (aggressive behavior)	Long-duration fight, chase, or threat between two individuals
Dust bath^a^	ATOL_0000824 (dustbathing)	Scrape and/or scrape the beak on the floor or litter, followed by vertical wing flaps, head rubbing, beak scraping, and or scraping with one leg in the extended position, and finally shakes to remove dust from the plumage
**Events behaviors**		
Comfort behaviors	ATOL_0000653 (comfort behavior trait) ATOL_0000822 (auto-body care trait) ATOL_0000823 (preening) ATOL_0000360 (body scratching)	Include five behaviors: Shaking: Ruffles the feathers and shakes the body Stretching: Stretches wing or leg Tail wagging: Wiggles its tail horizontally or vertically Wing flapping: Flaps its extended wings in a vertical plane Preening: Grooms its plumage with the beak in a sitting, lying, or standing position
Environment pecking	ATOL_0000845 (physical environment investigation)	Pecks something other than the ground, litter, feed, and water containers
Positive social pecking	ATOL_0000846 (conspecific investigation) ATOL_0000817 (approach response trait) ATOL_0000826 (allo-body care trait)	Pecks on the beak of a flockmate (mainly to collect food particles) or on another part of the body without damage to the plumage and skin of the targeted chicken

^a^These behaviors were rare (less than 5% of the observed individuals expressed the behavior) and were discarded from statistical analyses.

^b^Traits in reference to the ontology ATOL: https://www.atol-ontology.com/en/atol-2/.

Individuals had their behavior recorded over 16 days (6 days in period 1 and 5 days for period 2 and period 3), resulting in 32 min of observation per chicken.

### Ranging behavior measurements

**Figure 1:**
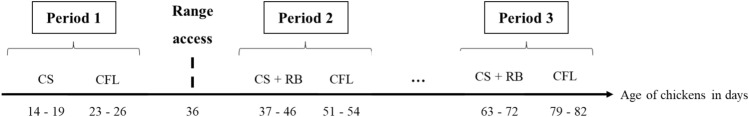
Schedule of the behavioral observations (Continuous sampling, CS, n = 58), Ranging behavior measurements (RB, n = 117), and contrafreeloading tests (CFL, n = 58) made during the production cycle of free-range chickens.

Measurements of chickens' ranging behavior levels followed the same procedures as described in^40–43^. Briefly, five interspaced scans per day (approximately 2 h between each scan, from sunrise to sunset) were performed during the same 10 days as per the behavioral observations after range access was allowed (Period 2 and 3, see Fig. 1), to determine the number of individual range visits by each of the 117 chickens (chickens carrying a poncho). The measurements of individual range visits of all 117 identified chickens (including the 58 chickens that had their behaviors observed and recorded) were done to have a larger continuum of range visits (from those who visit less the range to those who visit more), which would allow us to select a posteriori the most extreme individuals (see “Statistics”).

### Contrafreeloading test

Over three periods of 4 days each, before (period 1) and after (periods 2 and 3, see Fig. 1 for chickens' age) range access was made available, 58 individuals (the same individuals followed through continuous behavioral sampling) were tested for contrafreeloading in a controlled and individual test situation, using a conditioned place preference task, where animals were required to associate environmental cues with a particular stimulus^44–47^. Here the stimulus was either the presence of mealworms freely disposed on the floor (easy access to food) or the presence of mealworms mixed to a foraging substrate (difficult access to food).

The 58 chickens were divided into two subgroups of 30 and 28 individuals to be tested throughout the day (morning and afternoon, respectively). The test was carried out simultaneously in two identical rooms by two experimenters (approximately 15 individuals tested by one experimenter/half-day). The first subgroup was caught in the morning, taken to the testing room, and placed in a straw-covered enclosure with ad libitum water but no food for at least 2 h before the beginning of the task to standardize food motivation among individuals.

As soon as all the chickens from this subgroup finished their trials (a maximum of 3 h between the first and last individual tested), they were released back into the barn. In the afternoon, we proceeded in the same way with the second subgroup. The testing order of the individuals and the testing order of the subgroups were similar throughout the experiment.

**Figure 2:**
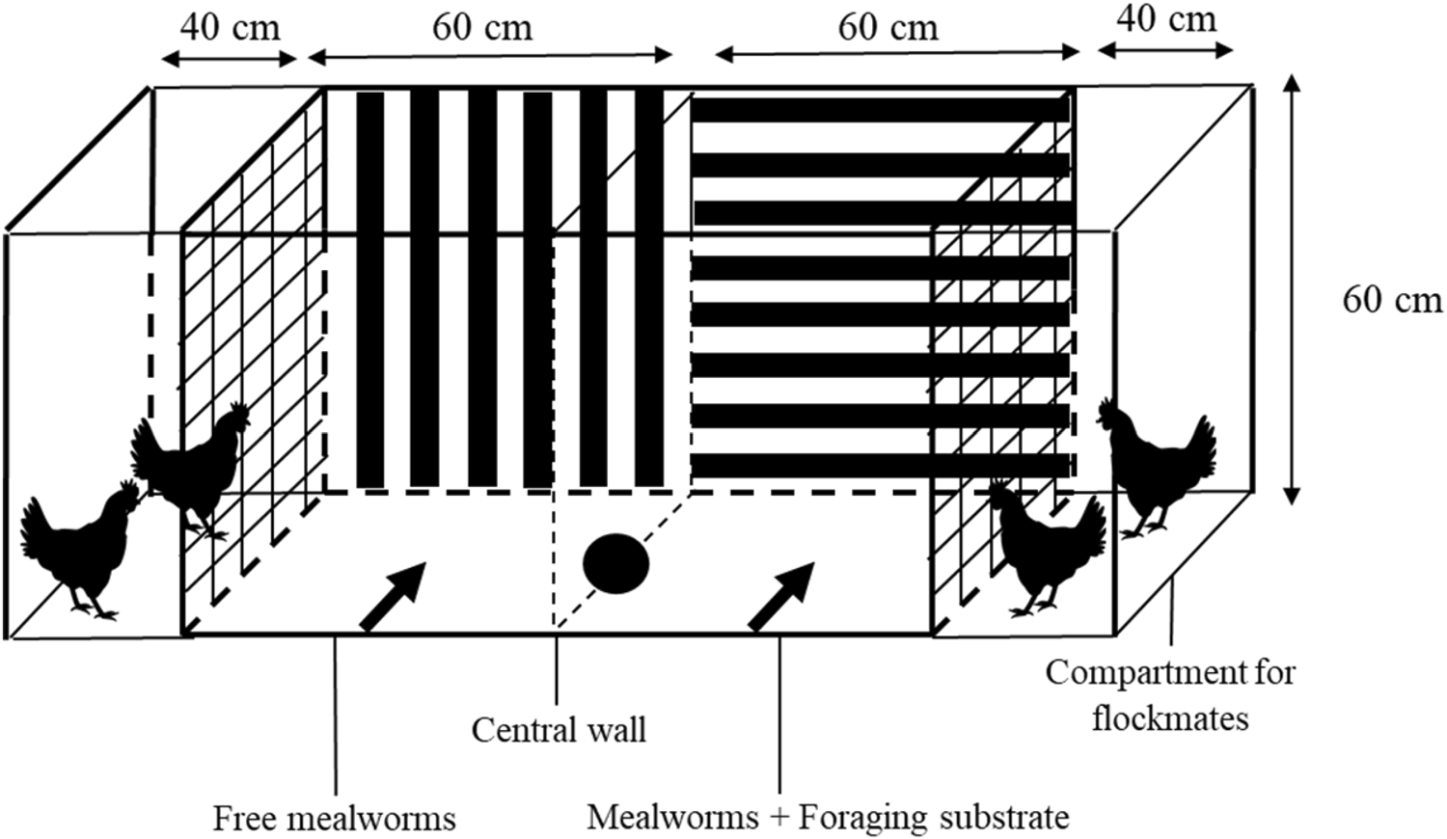
Side view of the arena (compartments and dimensions) used during the contrafreeloading conditioned place preference task (CPP). All over the task, and for all periods of testing, the extremes of each chamber were fenced to house two flockmate chickens (not tested). Chickens were always placed in the center of each chamber (conditioning, dark arrows) or the center of the apparatus when the wall was not present (test, dark circle).

The contrafreeloading test apparatus was a wooden structure with a yellow plastic floor, separated by a removable opaque wall into two chambers of 100 cm long each (Fig. 2). The walls of the two chambers were covered with different geometrical patterns. To prevent social isolation and stress, there was a fence allowing tested chickens to be in visual contact with two flockmates (reared in the same barn) within each chamber. Flockmates did not participate in the task. Flockmate pairs from both chambers were substituted for new ones at the end of the first round of individual trials. New pairs were chosen each day to prevent a social association to one side with preferred individuals.

To diminish any carryover effects between the periods, slight changes were made to the apparatus in its forms and patterns over the wall and the foraging substrate (see Fig. S1 in the “Supplementary material” for additional information).

#### Conditioning

During the first day of conditioning, individuals were placed in groups of four chickens in the arena and had access to only one chamber per trial (no free access to the two chambers) for 5 min twice a day (two trials). The time between each trial was approximately 1h20. One chamber contained free mealworms, and the other contained mealworms in a foraging substrate (straw, wood shavings, and hemp litter, according to the different periods). In the latter case, it would require an effort by the chickens for the mealworms to be obtained (contrafreeloading).

In each testing room, half of the chickens were conditioned to free food in one chamber and the other half in the other chamber to counteract a potential side bias on the animals' behavior. This order was then reversed for periods 2 and 3.

Over the following 2 days of conditioning, the animals were placed individually in the arena (body and head parallel to the central wall, always facing the long wall ahead), and similar to the group conditioning, they had access to only one chamber per trial for 3 min. 1h20 later, the individuals were placed in the other chamber of the apparatus. All animals ate and actively foraged for mealworms in all trials.

#### Test

We recently showed that free-range chickens exhibited, after only 4 conditioning days, a strong conditioned place preference during tests for a chamber where they could previously find food compared to a chamber with no food^43^. Here, after 3 days of conditioning animals to the two chambers, the test phase started. Individuals were placed in the center of the arena and had free access to both sides for two trials of 2 min and 30 s each. No mealworms nor foraging substrates were present in the arena. The purpose of this test is to subject individuals to the disappearance of food and foraging sources present in previous phases and to determine which side is preferred by chickens.

The variable recorded was the time spent in each of the chambers. It was observed directly by an experimenter outside of the view of the tested animal, using a digital video camera recorder connected to a monitor. The time spent on each chamber across the two trials was combined to calculate a preference index (PI) as follows:

$$PI=\frac{{T}_{FS}-{T}_{FM}}{{T}_{FS}+{T}_{FM}}$$

where T_FS_ is the time spent in the chamber with foraging substrate and T_FM_ the time spent on the chamber with freely accessible mealworms and without foraging substrate. This index could vary between 1.0 and 1.0. A positive value and significantly different from 0 indicated a preference for the chamber with a foraging substrate. Negative values indicated a preference for the chamber without a foraging substrate, where mealworms were freely disposed of on the ground.

## Statistics

We computed the activity budget (states) and frequency of behaviors (events) for each chicken through behavioral sampling. Each state behavior's duration was divided by the total time of observation, which gave us the time allocated in percentage. The total number of each event behavior was divided by the total hours of observation (32 min or 0.53 h per individual) to know the frequency of these behaviors (number of events/h).

Based on the number of individual range visits by each of the 117 chickens during period 2 (first 2 weeks of range access), we split all individuals into percentiles to select, among our 58 tested chickens, those falling in the first quartile (low values, indicating low ranging behavior) and individuals falling in the fourth quartile (high values, indicating high ranging behavior), these individuals were considered as the low- and the high ranger chickens, respectively. Out of our initial 58 observed and tested chickens, forty individuals were considered extremes: 19 low rangers (number of range visits = 1.05 ± 0.77) and 21 high ranger chickens (number of range visits = 6.61 ± 1.68) for subsequent analyses. The number of animals kept for statistical analyses in this study was based on a previous experiment, during which we measured range use and spatial memory^40^. By selecting, based on range use, 15 high rangers and 15 low rangers between 120 individuals, we obtained a difference between the two groups which was equivalent to 1 standard deviation of the trait and a statistical power of 79–83% depending on the test used (for p = 0.05). Taking the same difference between the two groups and a sample size of 20 animals per group increased the power to 86–91% depending on the test, which was more comfortable whether one or several behavioral data should be missing, due to individual death or exclusion from testing/observation during the whole production cycle.

As range use is more frequent towards the end of the production cycle due to habituation to the range^16,48,49^, we assumed early range use (period 2) is more linked to inter-individual differences in exploratory tendencies than late range use (period 3). Besides that, individual selection based on early range use is linked to cognitive processing between low and high ranger chickens^40–42^. Exploratory analyses showed that the number of individual range visits was significantly correlated across the periods (r_S_ = 0.367, p < 0.001, N = 117).

State behaviors and PI values were analyzed using a linear mixed model (LMM, 'lmerTest' R package), and degrees of freedom were estimated through the Satterthwaite approximation. When necessary, state behaviors were square-root transformed to meet normality assumptions. Ranging level (low and high rangers) and observation periods (1, 2, and 3) were added as fixed factors, and individual ID was considered a random factor to account for repeated observations. Exploratory analyses showed that the experimenter/experimental room did not influence PI values, neither as a main effect nor as an interaction with other fixed factors (all p > 0.05). This variable was, therefore, not included in the models. The interaction between the ranging level and period was not significant and was also excluded from all the final models. The normality of residuals was verified through the Shapiro–Wilk test and graphical evaluations (histograms and QQ plots). Marginal, conditional and semi-partial r-squared values (i.e., the proportion of variance explained by the fixed factors alone, by both random and fixed factors, and by each fixed effect adjusted for the other predictors in the model, respectively) of the final models were calculated and obtained using the r.squaredGLMM and r2beta functions (‘MuMIn’ and ‘r2glmm’ R packages)^50–52^. R-squared values range from zero to one, and can indicate small (0.02), medium (0.13) and large (0.26) effect sizes^53^. When main effects were significant, post hoc ANOVA comparisons of estimated marginal means ('emmeans' R package) were carried out with Tukey adjustment for multiple comparisons.

As event behaviors did not meet the assumptions for parametric statistics, even after transformations, non-parametric statistics were used. Friedman tests, followed by post-hoc Wilcoxon matched pairs test (Bonferroni-corrected), were used to examine any differences between periods, and Mann–Whitney U tests to examine differences between high and low rangers for each period and all periods combined. Kendall’s W and eta-squared values were also calculated to estimate the effect sizes of the non-parametric analyses^54,55^. Kendall’s W coefficient varies from zero (indicating no relationship between the dependent and independent variable) to one (indicating a perfect relationship), while the eta-squared values indicate small (0.01), medium (0.06) or large (0.14) effects^54,55^.

All statistical analyses were performed using IBM SPSS 21 and R v. 3.6.1. Statistical significance was accepted at p ≤ 0.05. Results are presented as raw mean ± standard error.

## Results

Six out of the eight behaviors analyzed were significantly influenced by the period of observation (cf. Table 2). While resting and feeding/drinking behaviors increased as animals aged, locomotion, foraging, comfort behaviors, and positive social pecking decreased over time. No effects of the period were seen for standing, nor environment pecking.

**Table 2:** Mean ± standard errors of state and event behaviors, on average, over the 3 periods (N = 40) or separately for low rangers (LR, N = 19) and high rangers (HR, N = 21). p-values (State behaviors and contrafreeloading: LMM Test; Event behaviors: Wilcoxon matched-pairs and Mann–Whitney U tests) and test statisticsa for all recorded behaviors. None of the interactions between fixed factors were significant and were excluded from the final models. r^2^m, r^2^c, and r^2^sp indicate the marginal, the conditional, and the semi-partial r-squared values, respectively, while η^2^ corresponds to the eta-squared values.

State behaviors		Period 1 (P1)	Period 2 (P2)	Period 3 (P3)	Statistics
Standing	Percentage (%)	29.9 ± 1.2	32.9 ± 1.4	28.4 ± 1.8	Period: F(2,78) = 2.7, p = 0.06
		LR: 28.8 ± 1.8	LR: 34.6 ± 2.1	LR: 26.4 ± 2.6	Ranging level: F(1,38) = 0.2, p = 0.63 r^2^m = 0.04; r^2^c = 0.12; r^2^sp (P2) = 0.01; r^2^sp (P3) = 0; r^2^sp (LR) = 0
		HR: 30.9 ± 1.7	HR: 31.2 ± 2	HR: 30.3 ± 2.5	
Resting		29.4 ± 1.6^a^	34.1 ± 1.7^a^	47.6 ± 2.4^b^	Period: F(2,78) = 26.2, p < 0.001
		LR: 32.3 ± 2.3	LR: 35.2 ± 2.5	LR: 50.9 ± 3.5	Ranging level: F(1,38) = 39, p = 0.053 r^2^m = 0.30; r^2^c = 0.37; r^2^sp (P2) = 0.02; r^2^sp (P3) = 0.26; r^2^sp (LR) = 0.03
		HR: 26.5 ± 2.2	HR: 32.9 ± 2.4	HR: 44.2 ± 3.3	
Locomotion		15.6 ± 0.7^a^	11.4 ± 0.7^b^	9.2 ± 0.6^b^	Period: F(2,78) = 24.4, p < 0.001
		LR: 15.4 ± 1.1	LR: 11.1 ± 1.1	LR: 9.6 ± 0.8	Ranging level: F(1,38) = 0, p = 0.98 r^2^m = 0.26; r^2^c = 0.34; r^2^sp (P2) = 0.13; r^2^sp (P3) = 0.26; r^2^sp (LR) = 0
		HR: 15.9 ± 1	HR: 11.7 ± 1	HR: 8.9 ± 0.8	
Foraging		18 ± 1.5^a^	8.6 ± 0.7^b^	1.7 ± 0.4^c^	Period: F(2,78) = 109, p < 0.001
		LR: 16.4 ± 2.2	LR: 5.5 ± 1	LR: 0.9 ± 0.6	Ranging level: F(1,38) = 10.6, p < 0.01 r^2^m = 0.62; r^2^c = 0.68; r^2^sp (P2) = 0.2; r^2^sp (P3) = 0.6; r^2^sp (LR) = 0.1
		HR: 19.5 ± 2.1	HR: 11.8 ± 1	HR: 2.6 ± 0.5	
Feeding/drinking		6.8 ± 0.8^a^	12 ± 1.3^b^	11.7 ± 1^b^	Period: F(2,78) = 8.1, p < 0.001
		LR: 6.7 ± 1.1	LR: 13 ± 1.8	LR: 10.8 ± 1.5	Ranging level: F(1,38) = 0, p = 0.8 r^2^m = 0.11; r^2^c = 0.16; r^2^sp (P2) = 0.08; r^2^sp (P3) = 0.09; r^2^sp (LR) = 0
		HR: 6.9 ± 1.1	HR: 11 ± 1.7	HR: 12.7 ± 1.4	
**Event behaviors**					
Comfort behaviors	Frequency (number/h)	29.1 ± 2.5^a^	16.9 ± 1.4^b^	19.5 ± 15^b^	Period: χ^2^(2) = 14,01 p = 0.001 Kendall’s W: 0.17
		LR: 29.1 ± 3.7	LR: 16.2 ± 2	LR: 18.3 ± 2.2	Ranging level: all p > 0.05 η^2^ (P1): 0; η^2^ (P2): 0; η^2^ (P3): 0
		HR: 29 ± 3.5	HR: 17.7 ± 1.9	HR: 20.7 ± 2.1	
Environment pecking		9.1 ± 1.8	8.6 ± 1.7	15.8 ± 2.6	Period: χ^2^(2) = 3.35, p = 0.18 Kendall’s W: 0.04
		LR: 8.7 ± 2.6	LR: 8.1 ± 2.5	LR: 11.8 ± 3.8	Ranging level: all p > 0.05 η^2^ (P1): 0.04; η^2^ (P2): 0.08; η^2^ (P3): 0.07
		HR: 9.5 ± 2.4	HR: 9 ± 2.4	HR: 19.9 ± 3.6	
Positive social pecking		18.3 ± 8.1^a^	5.5 ± 1^b^	5.8 ± 1.3^b^	Period: χ^2^(2) = 8.44, p = 0.01 Kendall’s W: 0.10
		LR: 8.7 ± 11.7	LR: 7.2 ± 1.4	LR: 4.9 ± 1.9	Ranging level: all p > 0.05 η^2^ (P1): 0.02; η^2^ (P2): 0; η^2^ (P3): 0.04
		HR: 27.9 ± 11.2	HR: 3.8 ± 1.4	HR: 6.7 ± 1.8	
**Contrafreeloading**					
Preference index		0.05 ± 0.05	0.05 ± 0.07	− 0.4 ± 0.8	Period: F(2,116) = 0.56, p = 0.57
		LR: − 0.06 ± 0.07	LR: 0.01 ± 0.11	LR: − 0.13 ± 0.13	Ranging level: F(1,116) = 4, p = 0.04 r^2^m = 0.04; r^2^c = 0.04; r^2^sp (P2) = 0; r^2^sp (P3) = 0; r^2^sp (LR) = 0.03
		HR: 0.17 ± 0.06	HR: 0.09 ± 0.1	HR: 0.05 ± 0.12	

^a^Different superscript indicate significant differences between periods.

**Figure 3:**
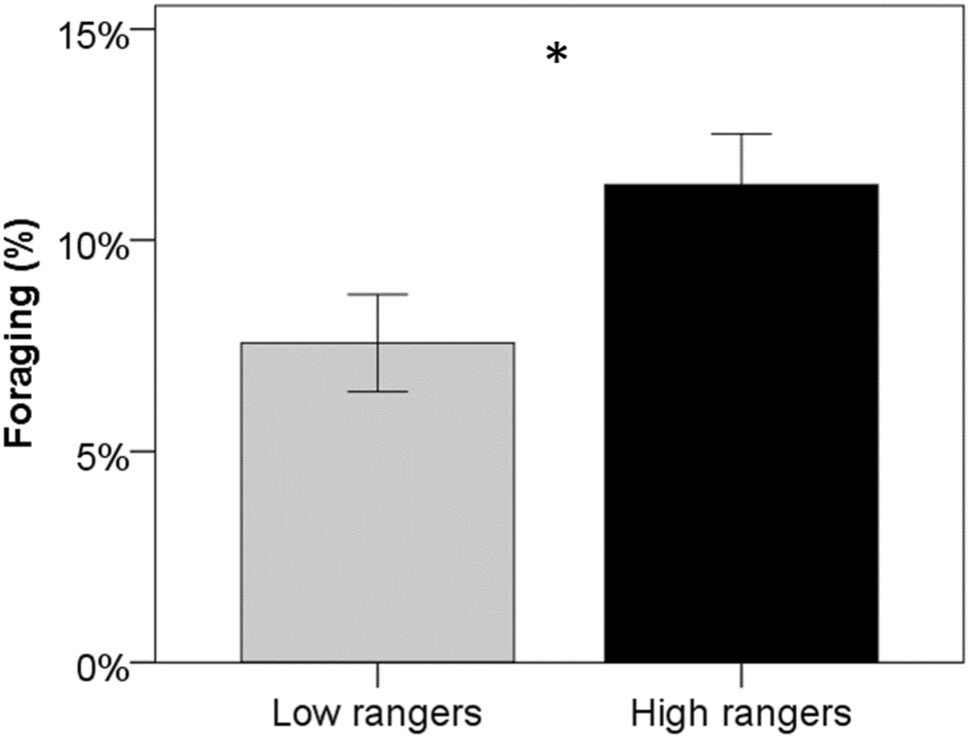
Percentage of time spent in foraging behavior averaged over three different periods of the production cycle (Period 1: before range access, Periods 2 and 3: after range access), for low (N = 19) and high ranger chickens (N = 21). *Differences between ranging groups at p < 0.05 (LMM Test). Data are presented as mean ± s.e.

Only foraging differed significantly between our two ranging groups. Independently of the period, low rangers foraged significantly less than high rangers (on average 7.5 ± 1.1% and 11.3 ± 1.2%, respectively, Fig. 3).

**Figure 4:**
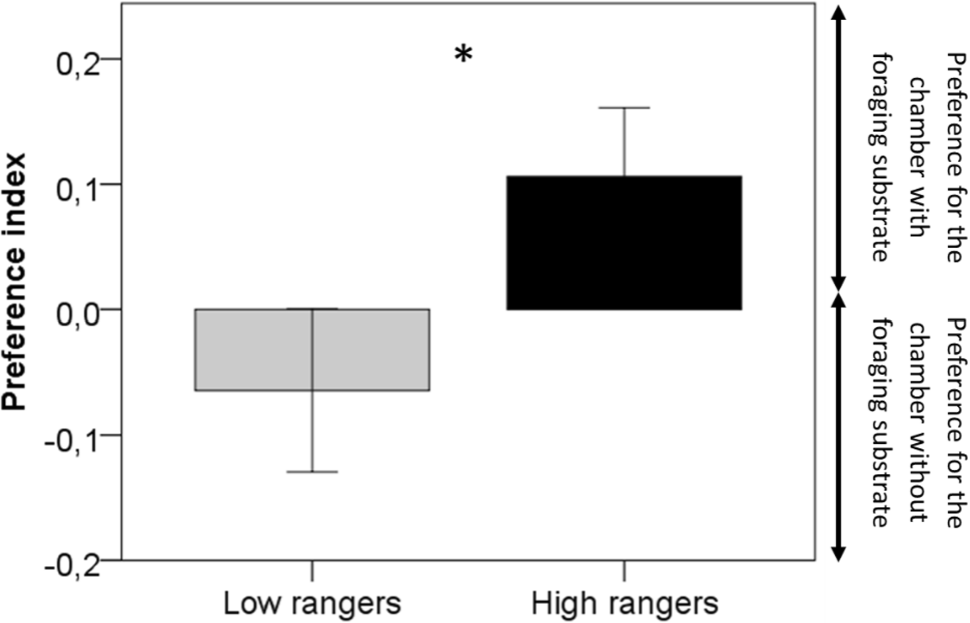
Preference index (PI) during a conditioned place preference task, averaged over three different periods of the production cycle (Period 1: before range access, Periods 2 and 3: after range access), for low (N = 19) and high ranger chickens (N = 21). The PI varied between 1.0 and 1.0. Positive values indicate a preference for the chamber with the foraging substrate, while negative values indicate a preference for the chamber where mealworms were freely disposed of on the ground. *Differences between ranging groups at p < 0.05 (LMM Test). Data are presented as mean ± s.e.

During the conditioned place preference task, the preference index was significantly influenced by ranging level, but not by period. Low rangers preferred the chamber where mealworms were easily accessible (without the foraging substrate), while high rangers preferred the chamber where mealworms were accessible with difficulty as it required contrafreeloading (Low rangers: − 0.06 ± 0.06, High rangers: 0.1 ± 0.05, Fig. 4).

## Discussion

Using the free-range broiler chicken as a model species, our findings allowed us to establish a link between the contrafreeloading phenomenon and chicken inter-individual differences, in this case, the ranging behavior. High rangers were more motivated to make efforts to obtain food than their low ranger conspecifics during an individual contrafreeloading test. Besides that, differences in ranging behavior were also associated with differences in chickens' motivation to forage, consistent with contrafreeloading test results. More interestingly, these differences were significant for all periods, even before range access, when animals are still in the barn, suggesting a potential predictor sign of range use. The observation period significantly impacted most of the studied home environment behaviors since chickens either increased the time spent or the frequency of some behaviors (resting and feeding/drinking) but reduced other activities (foraging and locomotion). Chickens were less prone to forage over time, which suggests a natural reduction in their willingness to contrafreeload.

Our first two main objectives in this work were to evaluate free-range chickens' contrafreeloading in two situations: individual testing and individuals' home environment through their foraging. Our results suggest that contrafreeloading and foraging share similarities and may share, in fact, the same behavioral basis, as suggested by a previous work^17^. They were the only behaviors that differed between low and high ranger chickens, even though these behaviors were measured in different situations. Furthermore, the fact that free-range broiler chickens gradually diminished their time on demanding behaviors, such as foraging and locomotion, and allocated more time to less costly behaviors, such as resting and maintenance behaviors, is in accordance with the theory of resource allocation^6,38^. A similar pattern occurred for sexually mature junglefowl and white leghorn fowl, in which contrafreeloading decreased with age when resources have to be dedicated to reproduction^38^. Another possibility for this reduction in foraging with age is that older animals need more food to support their metabolic needs and may become hungrier, which is supported by the increase in feeding/drinking behaviors. Studies have shown that there is a negative relationship between hunger levels and contrafreeloading^5^. For example, starlings (*Sturnus vulgaris*) subjected to four periods of food deprivation (0 h, 2 h, 4 h, 8 h) reduced their contrafreeloading as deprivation time increased. However, even during the most prolonged deprivation period, animals still acquired 25% of their consumption through searching for hidden food instead of eating freely visible food^56^, confirming a strong motivation to acquire food through this specific way.

It is interesting to note that, although feeding/drinking behaviors increased over periods for both groups, there were no differences between low and high rangers, suggesting that, similarly to cattle^37^ and pigs^2^, foraging and contrafreeloading behaviors are unrelated to the individual satiation level: low and high rangers have similar appetites. These results point towards two possible hypotheses, some individuals forage and contrafreeload to acquire more information ('information primacy' model), or these behaviors are behavioral needs that are rewarding per se (self-reinforcing theory). To test the self-reinforcing theory, for example, it would be interesting to expose chickens to one chamber containing a foraging substrate (but no food) while the other chamber would be empty. If the foraging substrate per se, without any food reward, is capable of eliciting contrafreeloading in high rangers, this could suggest that contrafreeload occurs mainly because foraging behaviors are self-rewarding and important behavioral needs for this group of animals. More studies need to be done to disentangle these two hypotheses in free-range chickens.

Unlike foraging behavior under the home environment condition, the preference index during the contrafreeloading test did not vary significantly over periods. This result can be explained by the fact that the test situation did not request animals to actively search for food (the test arena being empty of mealworms and foraging substrates), as it was the case for the foraging behavior in the range/barn. Therefore, it is possible that the costs of showing contrafreeloading during the tests were lower than those of a real foraging situation, which may have caused individual responses to be more stable over periods. It is also important to mention that, due to their increased experience with their home environment over different periods, chickens may have acquired sufficient information on the available food sources, which may have reduced their motivation to contrafreeload (in the form of foraging) over time. This increased experience was lowered in the individual testing situation due to the time elapsed between each testing period and modifications done to the arena (shape, cues on the walls, and foraging substrates). Another possibility for the differences may rely on the social environment differences between the contrafreeloading in the range/barn and contrafreeloading in the test situation. Chickens are social animals and are influenced by the behavior of their conspecifics^18^. While in the home environment, chickens may be influenced by the foraging of other individuals. In the test situation, tested individuals had limited social contact with two conspecifics through a fence. These flockmates did not have mealworms or foraging substrates and could not perform, therefore, any eating or foraging. In a similar situation, junglefowl performed higher contrafreeloading levels when tested in pairs compared to when tested individually^38^.

It is important to consider that our results during the contrafreeloading test may also be interpreted as animals exhibiting a simple preference for the foraging substrate: high rangers may be, for example, more sensitive and prefer to walk on substrates, compared to walking on bare floors, and therefore, prefer the chamber associated to them. However, this explanation is unlikely since the preference of high rangers for the chamber with foraging substrate did not seem to depend on the substrate presented. Indeed, in our experience, three very different substrates (from a visual, tactile, and olfactory point of view) were used during the three test periods. Despite this, high rangers consistently spent more time in the chamber with foraging substrate during the three test periods. Furthermore, our results cannot be explained by an immediate preference of high rangers to explore or forage on the substrates since additional analyses showed that, during conditioning, high and low rangers spent the same amount of time foraging in the substrates (period effect: p = 0.16; ranging level effect: p = 0.40; r^2^m = 0.08; r^2^c = 0.26; r^2^sp (P2) = 0; r^2^sp (P3) = 0.04; r^2^sp (LR) = 0.02). During the test, the foraging substrates were no longer present in the arena, and the preference expressed by the high rangers cannot, therefore, be explained by a sensorial preference for the substrate. Our results suggest that, during the test, the preference of high rangers for the chamber with a substrate is mainly mediated by cognitive processes linked to how individuals perceived and memorized the situation encountered. In line with this, our previous work on different cognitive aspects of the free-range broiler chickens strongly suggests that their differences are related to how they perceive their environment cognitively, with no signs of sensorial differences (smell or visual, for example) between high and low rangers during learning/conditioning phases. Differences occurred mainly when a food reward was no longer present^40,42^ or presented differently^41^ compared to learning/conditioning phases when food reward was always present. Finally, previous work on contrafreeloading of junglefowl and laying hens showed that even when tested in their pens with free access to a substrate (wood shavings) during tests, junglefowl had a greater preference to feed in food sites where food was difficultly accessible compared to laying hens^8^. The same occurred for laying hens compared to broilers chicks^6^. Considering all these elements, these arguments lean towards the idea that high rangers are really motivated for foraging, and therefore for contrafreeloading, both in individual and group situations, as confirmed by foraging results in the home environment condition.

Our third and final objective was to check whether differences between our ranging groups were specific to contrafreeloading and foraging behaviors. We found that there was no support for other behavioral differences between these groups despite a trend for differences in resting behavior between high and low rangers. Our results corroborate that ranging behavior is mainly linked to foraging and contrafreeloading, and these behaviors are probably part of a large exploratory personality trait of free-range chickens. The exploratory trait is known to modulate, among other things, the way individuals perceive and interact with their physical and social environment^57,58^. We recently showed that low rangers have better cognitive abilities on multiple tasks, such as spatial and non-spatial memory task^40,42^, and cognitive flexibility^41^. All these results combined suggest the existence of a trade-off between speed and accuracy, as suggested by Sih and Del Giudice^59^. Compared to low rangers, high rangers may explore novel environments quicker but may ignore the environmental cues, which causes them to be more impulsive and poor learners.

This work aimed to bring new empirical elements to advance biological theories of individual differences in exploratory ranging behavior, foraging, and contrafreeloading. High and low rangers differ in their motivation to forage, and contrafreeload and these differences appear even before they can access the range. The study of farm animals' individual differences brings new elements to fundamental questions that are still open to discussion and allows a better knowledge of the peculiarities of the individuals composing a group, laying the foundation for improving animal welfare. Since differences in foraging and contrafreeloading can be seen before range access, future studies need to focus on early life conditions to increase group foraging and motivate all individuals to use the range more. Increasing the knowledge of individual particularities is a necessary step to improve free-range chicken welfare on the farm.

## Supplementary information

Supplementary information.

Supplementary figure S1.
