# Supplementary material for: Working for food is related to range use in free-range broiler chickens
Source: Sci Rep. 2021 Mar 18;11:6253. doi: 10.1038/s41598-021-85867-2 (PMC7973526; doi:10.1038/s41598-021-85867-2)
Supplement: Supplementary file 2 — Supplementary figure S1. [file 41598_2021_85867_MOESM2_ESM.docx]

Electronic supplementary material

**Working for food is related to range use in free-range broiler chickens**

Vitor Hugo Bessa Ferreira, Arthur Simoni, Karine Germain, Christine Leterrier, Léa Lansade, Anne Collin, Sandrine Mignon-Grasteau, Elisabeth Le Bihan-Duval, Elodie Guettier, Hélène Leruste, Ludovic Calandreau, Vanessa Guesdon


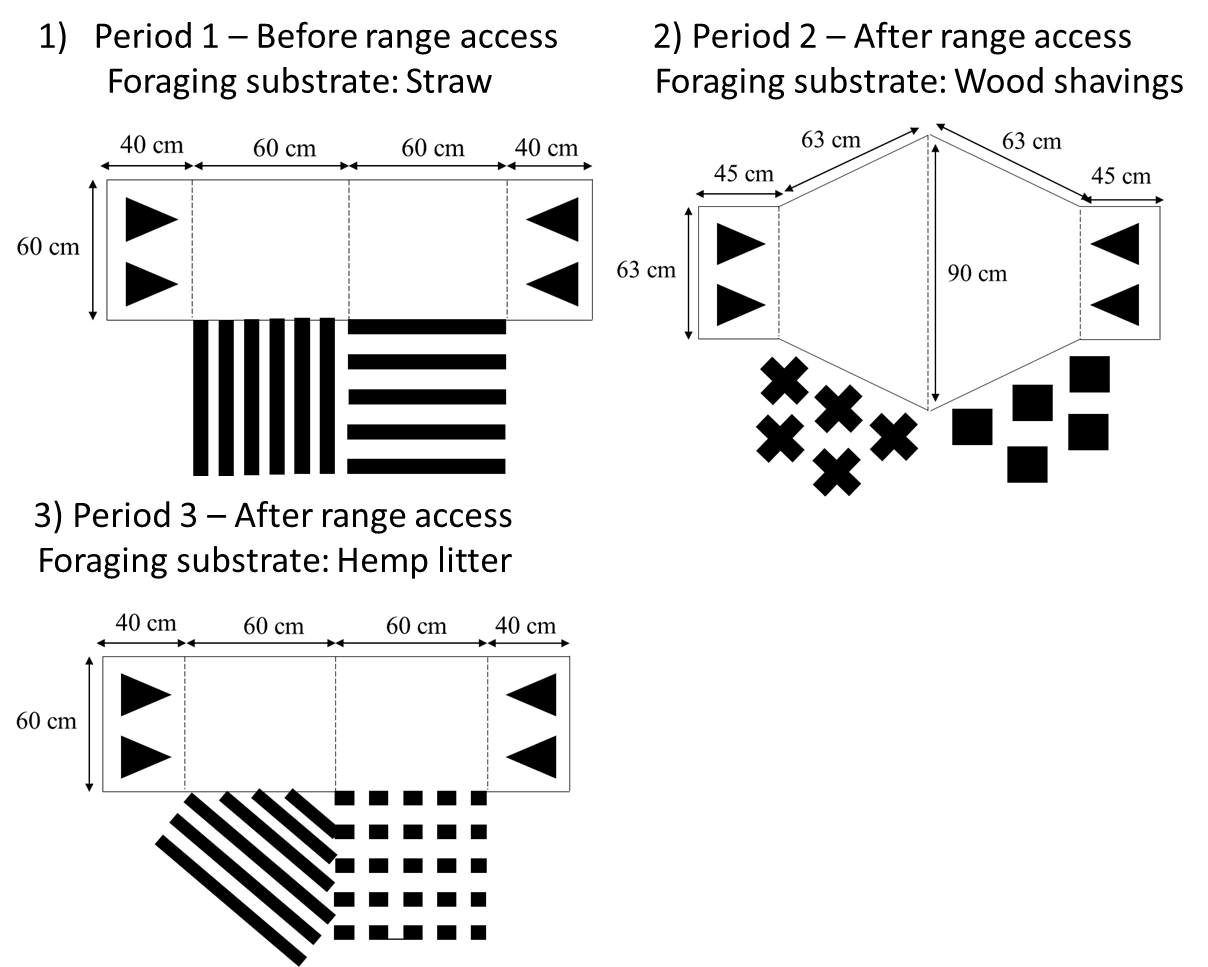


Figure S1: Schematic figure of the apparatuses used during the contra-freeloading conditioned place preference task (CPP). Each chamber had different patterns on the wall, represented by different geometric black patterns outside the rectangle. One chamber contained free mealworms, and the other contained mealworms in a foraging substrate (straw, wood shavings, and hemp litter, according to the different periods). During all the task (conditioning and test), each chamber's extremes were fenced to house two flockmate chickens (black triangles). Tested chickens were always placed near the central wall (conditioning) or in the apparatus's center when the wall was not present (test).
